# Spatial clustering of patent and sub-patent malaria infections in northern Namibia: Implications for surveillance and response strategies for elimination

**DOI:** 10.1371/journal.pone.0180845

**Published:** 2017-08-18

**Authors:** Jennifer L. Smith, Joyce Auala, Munyaradzi Tambo, Erastus Haindongo, Stark Katokele, Petrina Uusiku, Roly Gosling, Immo Kleinschmidt, Davis Mumbengegwi, Hugh J. W. Sturrock

**Affiliations:** 1 Malaria Elimination Initiative, Global Health Group, University of California San Francisco, San Francisco, California, United States of America; 2 Multidisciplinary Research Center, University of Namibia, Windhoek, Namibia; 3 National Vector-Borne Disease Control Program, Ministry of Health and Social Services, Windhoek, Namibia; 4 MRC Tropical Epidemiology Group, Department of Infectious Disease Epidemiology, London School of Hygiene and Tropical Medicine, London, United Kingdom; Université Pierre et Marie Curie, FRANCE

## Abstract

**Background:**

Reactive case detection (RACD) around passively detected malaria cases is a strategy to identify and treat hotspots of malaria transmission. This study investigated the unproven assumption on which this approach is based, that in low transmission settings, infections cluster over small scales.

**Methods:**

A prospective case-control study was conducted between January 2013 and August 2014 in Ohangwena and Omusati regions in north central Namibia. Patients attending health facilities who tested positive by malaria rapid diagnostic test (RDT) (index cases) were traced back to their home. All occupants of index case households (n = 116 households) and surrounding households (n = 225) were screened for *Plasmodium* infection with a rapid diagnostic test (RDT) and loop mediated isothermal amplification (LAMP) and interviewed to identify risk factors. A comparison group of 286 randomly-selected control households was also screened, to compare infection levels of RACD and non-RACD households and their neighbours. Logistic regression was used to investigate spatial clustering of patent and sub-patent infections around index cases and to identify potential risk factors that would inform screening approaches and identify risk groups. Estimates of the impact of RACD on onward transmission to mosquitoes was made using previously published figures of infection rates.

**Results:**

Prevalence of *Plasmodium falciparum* infection by LAMP was 3.4%, 1.4% and 0.4% in index-case households, neighbors of index case households and control households respectively; adjusted odds ratio 6.1 [95%CI 1.9–19.5] comparing case households versus control households. Using data from Engela, neighbors of cases had higher odds of infection [adjusted OR 5.0 95%CI 1.3–18.9] compared to control households. All infections identified by RDTs were afebrile and RDTs identified only a small proportion of infections in case (n = 7; 17%) and control (0%) neighborhoods. Based on published estimates of patent and sub-patent infectiousness, these results suggest that infections missed by RDTs during RACD would allow 50–71% of infections to mosquitoes to occur in this setting.

**Conclusion:**

Malaria infections cluster around passively detected cases. The majority of infections are asymptomatic and of densities below the limit of detection of current RDTs. RACD using standard RDTs are unlikely to detect enough malaria infections to dramatically reduce transmission. In low transmission settings such as Namibia more sensitive field diagnostics or forms of focal presumptive treatment should be tested as strategies to reduce malaria transmission.

## Introduction

Reactive case detection (RACD) is a widely used surveillance method in low endemic and elimination settings, in which household members and neighbours of passively detected cases (index cases) are tested and treated when positive [1–3]. The rationale for RACD is based on the spatial characteristics of malaria transmission, which becomes increasingly focal and clustered into geographical hotspots as it declines [2]. These hotspots may be single or groups of households which experience higher levels of transmission relative to others in the community. Targeting screening and interventions to these higher-risk households is a cost-effective alternative to blanket strategies, provided that programmes know where and over what scale hotspots exist [4].

Despite the intuitive appeal and increasing programmatic uptake of RACD, the evidence base supporting the potential impact of this strategy is inconsistent. RACD around the households of index cases has been shown to be an effective method to identify additional asymptomatic infections in some contexts, including Zambia [3] and Swaziland [1]. In Swaziland, additional infections were more likely to be found in the household of index cases than in neighbouring households, illustrating the highly clustered nature of malaria transmission in this setting. However, RACD is unlikely to be effective as an intervention in settings where transmission occurs away from the place of residence, such as forest-fringe areas of Cambodia [5] and India [6].

The potential impact of RACD is further undermined by the widespread use of rapid diagnostic tests (RDTs) as a point-of-case diagnostic, due to their low sensitivity for low-density infections [7]. An increasing body of evidence suggests that RDTs miss a substantial proportion of low density infections even in low endemic settings, compared to molecular diagnostic techniques [8, 9]. While sub-patent infections are likely to be less infectious than high density parasitaemia, they are still able to infect mosquitoes [7] and due to potentially large numbers, contribute substantially to the infectious reservoir [10]. Screen and treat strategies, particularly in settings where high proportions of infections are asymptomatic, may therefore require a more sensitive diagnostic to impact transmission [11]. Further evidence around the operational effectiveness of RACD in different settings is needed to assist decision makers and support initiatives to improve diagnostics.

This study investigated: (i) the detection rate of RDT based RACD compared with RACD based on more sensitive molecular diagnostics; (ii) spatial clustering of infections around passively determined index cases; and (iii) risk factors for secondary infection in a low transmission setting in northern Namibia.

## Methods

### Data collection

**Fig 1:**
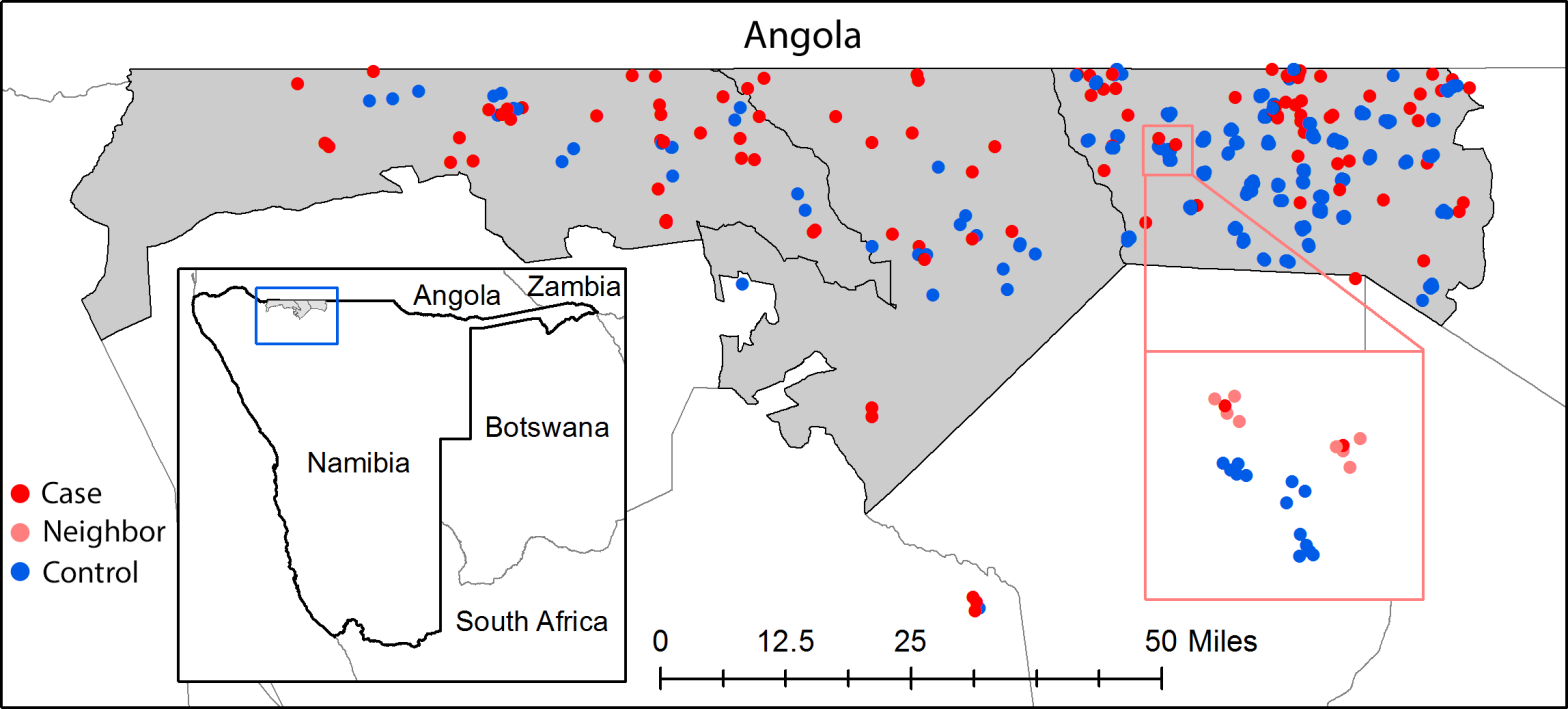
Location of index case (red) and control (blue) households within Engela, Outapi and Oshikuku health district within Namibia. Inset map shows a zoomed view of neighboring households (coral) screened around index case households.

This study is part of a broader investigation of the epidemiology of malaria in Ohangwena and Omusati regions in north central Namibia, which included a case-control study [12]. In this setting, all malaria infections are due to *P*. *falciparum* which was confirmed by speciation by PCR [13]. Between January 2013 –August 2014, passively-detected RDT confirmed cases of malaria presenting to any public health facility within Engela, Outapi and Oshikuku health districts were followed up to their home (Fig 1). While the study aimed to conduct RACD within a 48 hour window from index case diagnosis, in keeping with national guidelines, in practice only 50% of investigations occurred within a two week time frame. Follow up occurred up to two months after diagnosis when case burden was exceptionally high. Index cases along with all other household members were screened by RDT and interviewed using a standard questionnaire. A dried blood spot (DBS) was collected for later molecular analyses from participants in Engela district and a subset of households in Outapi and Oshikuku. Members of the four nearest households neighbouring the index case, up to a maximum of 30 individuals, were recruited in Engela district. This threshold was selected based on the expected population per household. Due to resource constraints, RACD was not implemented around index case and control households in Outapi and Oshikuku.

The same data (RDTs and DBS) were collected from randomly selected ‘control’ households, frequency matched to controls by district [12], using the 2011 census. As for case households, the four nearest neighbouring households of each ‘index’ control household in Engela were also recruited to participate in the study and included as controls.

If individuals were not present in the selected households, study teams made two return visits to complete data collection. If unsuccessful after the third attempt, the household was replaced by the nearest eligible neighbouring household. A standard questionnaire was used to collect data for all participants, providing information on demographics, relationship to household, and travel history. Interviews were conducted in Oshiwambo. Household location coordinates in decimal degrees were captured using tablets (Google Nexus 7) with built-in global positioning systems (GPS) and used to calculate distance from the index case household. The majority (93%) of neighbors screened during RACD were within 500m of the index case household.

Written informed consent was provided by all participants after explanation of the rationale and procedures of the study. Consent of a parent or guardian was required for those younger than age 18 years. Individuals testing positive for malaria by RDT were treated according to standard national guidelines with artemether/lumefantrine combination therapy by registered nurses accompanying field teams.

### Ethical approval

Institutional Review Board approval for this study was obtained from the University of Namibia, the Ministry of Health and Social Services of Namibia, the University of California San Francisco, USA and the London School of Hygiene and Tropical Medicine, UK.

### Laboratory analyses

Laboratory procedures and a full comparison of LAMP as a surveillance tool in comparison to nested-PCR is described in Tambo et al [13]. In brief, DNA was extracted from DBS samples using chelex extraction method and processed using Pan-LAMP tubes (LMC 562, Eiken Chemical Co., Ltd. Tokyo, Japan), which detect all four species of Plasmodium. Results were compared against RDT results used in the field to determine the infection status of individuals for treatment at the time of screening.

### Statistical analyses

For the purposes of this study and following conventional terminology [14], we refer to a passively detected case as an ‘index case’. All additional infections diagnosed with malaria during screening, in this case using LAMP, are hereafter termed “secondary infections” to distinguish them from the index case. Index case households are those households in which index cases reside, while neighboring households are those screened around the index case household during RACD (in Engela only). Individuals with missing or invalid RDT or LAMP results were excluded from all analyses.

Namibia does not routinely classify cases as autochthonous (local) or imported and all index cases residing within the study area were followed up for RACD. For the purpose of this analysis, we classified index cases as imported based on reported travel outside of Namibia within the previous six weeks. Distance of neighboring households to the index case household in kilometers was calculated from decimal degree coordinates using the ‘gmt’ package in R 3.3.1 [15]. Guided by the total burden of passive case detection, cases between January and May were classified as being in the high season and those detected June to December as low season. Time to follow up was calculated as the difference in days between the date of diagnosis of the index case and date of RACD screening.

To examine clustering of infection around passively detected cases, two analyses were done. First, the probability of infection (as determined by LAMP) in non-index individuals living in neighbourhoods of cases was compared to individuals in control neighbourhoods using logistic regression. Given the clustered nature of the data (i.e. individuals within households), a household level random effect was included to adjust for correlation between individuals within the same household. Second, using only data from case neighbourhoods, the scale of spatial clustering of secondary infections was examined by exploring the relationship between distance to index case and probability of detecting an infection. As with the first analysis, logistic regression was used with a household level random effect. Distance to index case household was compared as a linear, quadratic and binary (inside versus outside the household of the index case) term using a likelihood ratio test. The unadjusted and adjusted odds of infection were calculated in relation to time to follow up, characteristics of the index case (gender, age, if case was in low or high transmission season, if case was imported, if the index case slept under a mosquito net the previous night and if index house had been sprayed in the past year and individuals screened during RACD (gender, age, travel to Angola in the past 30 days, slept under a mosquito net the previous night and if house had been sprayed in the past year). Statistical analyses were conducted using STATA 12.1 [15] and R 2.12 [14].

To assess the prevalence of patent and sub-patent infections, RDT results were compared to LAMP as a gold standard. A parallel study showed excellent concordance between LAMP and duplicate nPCR [13]. To estimate the impact that RACD using RDTs would have on transmission, data on infectiousness of patent and sub-patent infections was taken from Okell et al. (2012) [16] and used to calculate the proportion of infections to mosquitos that would be averted after correcting for the higher false positivity rate of RDTs compared to microscopy [17]. For the purpose of this analysis, it was assumed that 100% of infections would be averted if all LAMP positives were treated. For these analyses only data from case neighbourhoods was used, as this reflects the operational context of RACD.

## Results

### Reactive and population screening

Between December 2012 –July 2014, 146 malaria cases were passively identified by RDT in the study area and their households visited. Based on available data in Engela, this represents 50.7% of malaria cases eligible for follow-up based on their residence in the study area. Results from the case-control study are presented in a separate publication [12], while this paper will focus on reactive screening and clustering of secondary infection. Index case households (n = 127) and neighboring houses in Engela (n = 246) were surveyed, as well as 373 households in control neighborhoods. In case neighborhoods, 9.8% (n = 228) of individuals were absent at the time of initial screening and return visits compared to 12.1% (n = 249) in control neighborhoods (p = 0.02). Individuals missing LAMP or RDT results were further excluded in case (n = 252; 12.0%) and control (n = 516; 28.5%) neighborhoods (p<0.0001). A third of missing laboratory samples (35.5%) were attributed to lack of consent, which accounted for 7% (n = 273) of all individuals present at the time of survey.

In total, 3,151 individuals were successfully screened using DBS and RDT; 1,856 individuals from case households (n = 116) and their neighbors (n = 225) and 1,295 individuals from control households (n = 286) (Table 1). Characteristics of individuals in case households differed from neighboring and control households (Table 2): they were more likely to be male, aged between 15–24 years and have reported travel to Angola. Individuals in case households were also less likely to report IRS in the past year or have slept under a bednet the previous night.

**Table 1:** Number of participants screened in case, neighboring and control households in Engela, Outapi and Oshikuku health districts.

	Number screened			
	Engela	Outapi	Oshikuku	Total
Index-case households	58	38	20	116
Participants	509	209	83	801
Neighboring households	225	0	0	225
Participants	1,055	0	0	1,055
Control households	250	22	14	286
Participants	1,077	175	43	1,295

**Table 2:** Characteristics of individuals screened for malaria in case and control neighborhoods by RDT and LAMP.

		Number (%)			
		Index-case households (N = 116)	Neighbors of case households^2^ (N = 225)	Control households (N = 286)	p-value^3^
Number individuals^1^		801	1,055	1,295	-
RDT positive		11 (1.4)	7 (0.7)	5 (0.4)	0.03
LAMP positive		27 (3.4)	15 (1.4)	5 (0.4)	<0.0001
Sub-patent^4^		21 (2.6)	14 (1.3)	5 (0.4)	<0.0001
Gender	Female	376 (46.9)	634 (60.1)	730 (56.4)	<0.0001
	Male	425 (53.1)	421 (39.9)	565 (43.6)	
Age category (years)	<5	108 (13.5)	181 (17.2)	214 (16.5)	<0.0001
	5–14	187 (23.3)	324 (30.7)	405 (31.3)	
	15–24	289 (36.1)	226 (21.4)	209 (16.1)	
	25–34	83 (10.4)	99 (9.4)	121 (9.3)	
	35–44	54 (6.7)	66 (6.3)	101 (7.8)	
	45+	76 (9.5)	152 (14.4)	211 (16.3)	
	Missing	4 (0.5)	7 (0.7)	34 (2.6)
Reported fever		30 (3.7)	62 (5.9)	100 (7.7)	0.003
	Missing	0 (0.0)	2 (0.2)	4 (0.3)
Season	Low	127 (15.9)	255 (24.2)	645 (49.8)	<0.0001
	High	674 (84.1)	800 (75.8)	650 (50.2)	
Travelled to Angola		33 (4.1)	24 (2.3)	14 (1.1)	<0.0001
Used a bednet		153 (19.1)	294 (27.9)	298 (23.0)	<0.0001
	Missing	9 (1.1)	7 (0.7)	12 (0.9)	
House sprayed		202 (25.2)	304 (28.8)	426 (32.9)	0.01
	Missing	57 (7.1)	15 (1.4)	9 (0.7)

RDT: rapid diagnostic test; LAMP: loop mediated isothermal amplification

^1^Excluding index cases

^2^ Engela only

^3^ Pearson's Chi-squared test

^4^ RDT negative and LAMP positive

### Clustering and risk factors for secondary infection

**Fig 2:**
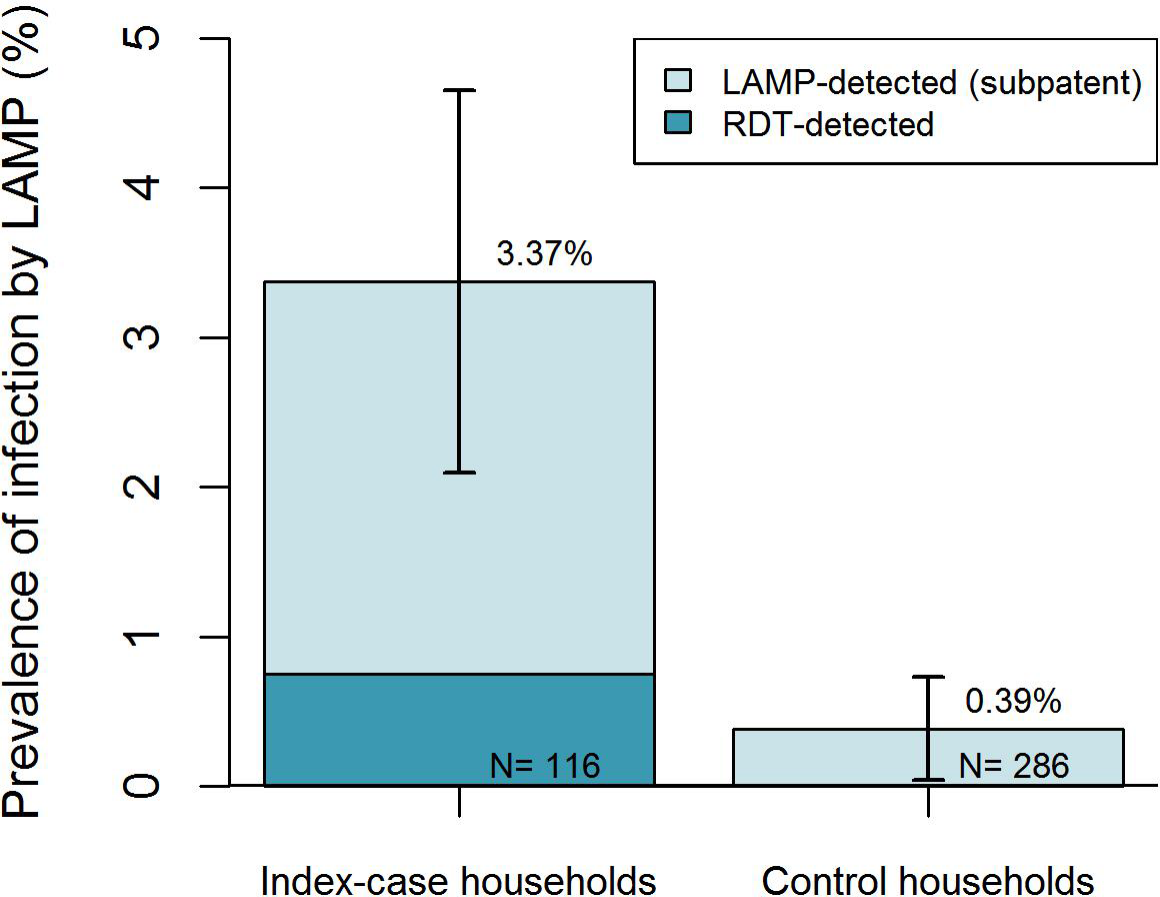
Prevalence of patent and sub-patent secondary infections in index case households and control households in Engela, Oshikuku and Outapi districts.

Overall, the prevalence of secondary infection by LAMP was higher in case households (3.4%) compared to control households (0.4%) (Table 2; Fig 2). The odds of secondary infection remained six-fold higher in case households compared to control households (OR 6.1 95%CI 1.9–19.5), after accounting for household clustering and controlling for differences in the transmission season (high vs low). In addition, there was weak evidence that males had a lower odds of being infected in case households (OR 0.4 95%CI 0.1–1.0). This may be due to the exclusion of index cases from the screened population (who were more likely to be males themselves [12]) or potential bias arising from absenteeism, given that those absent during screening were more likely to be male compared to those screened (58.4% vs 44.4%; p<0.0001).

**Fig 3:**
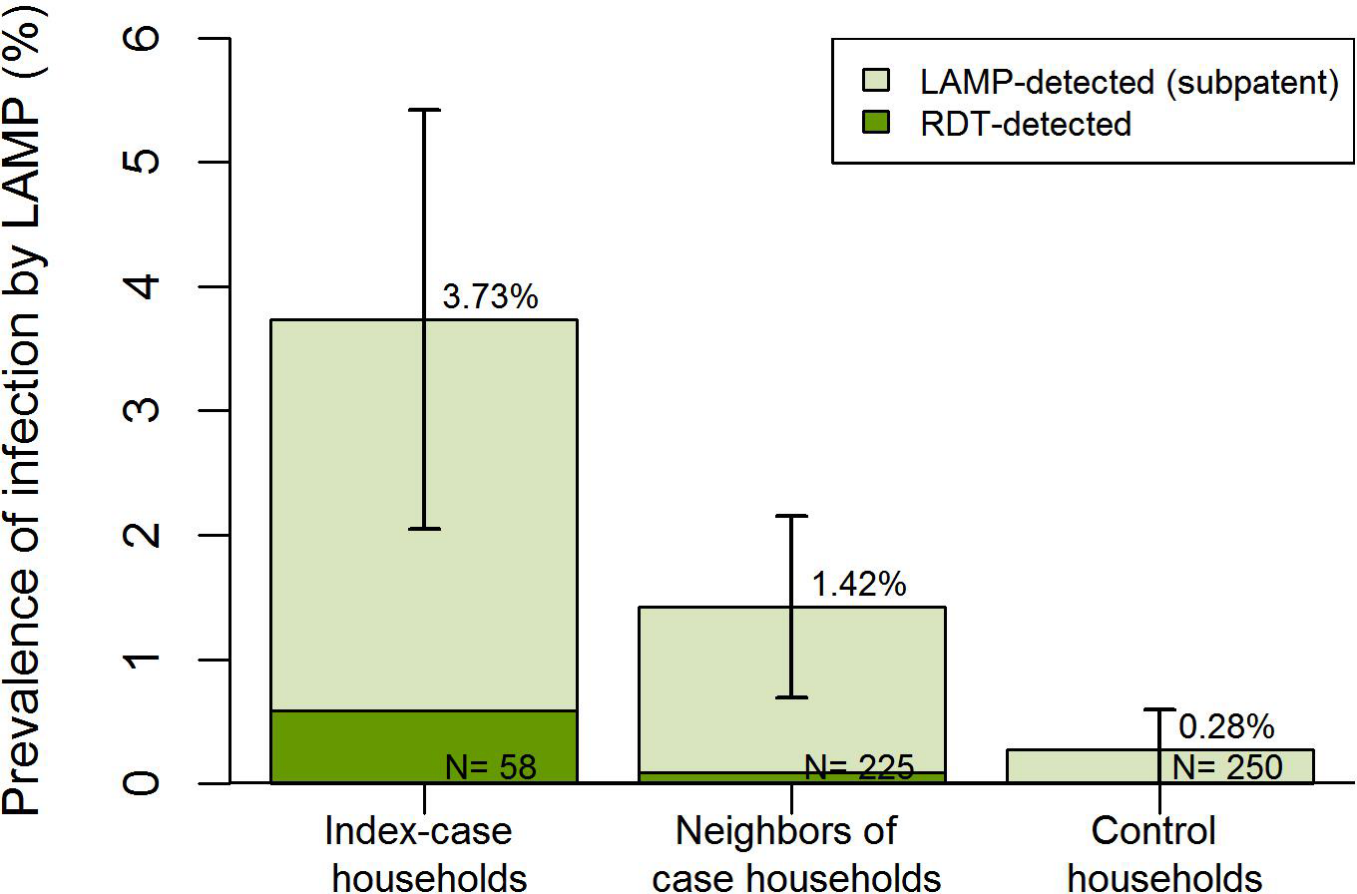
Clustering of patent and sub-patent secondary infections around passively detected index cases in Engela, compared to control households.

In Engela, there were higher odds of secondary infection both in case households (OR 9.9 95%CI 2.3–43.1) and neighbors of case households (OR 5.0 95%CI 1.3–18.9) compared to control households (Fig 3), after accounting for within-household correlation. Focusing on the data from case neighbourhoods only, while there was an increase in the prevalence of infection in index case households compared to their neighbours, this was not statistically significant (OR 1.39 95%CI 0.5–3.6) and there was no evidence of spatial decay of risk (Table 3). The adjusted odds of finding a secondary infection were higher during the higher season (OR: 4.07 95%CI: 1.4–11.7), while they were lower in neighborhoods of index cases that reported sleeping under a bednet the prior night (OR: 0.04 95%CI: 0.01–0.9) (Table 3). There was no evidence of an association between secondary infections and other characteristics of passively detected cases or individuals screened in case neighborhoods (Table 3).

**Table 3:** Characteristics of 1,564 individuals screened in case and neighbours of case households during RACD in Engela.

Characteristics		Numbers positive / numbers examined (%)	Unadjusted		Adjusted	
			OR	95% CI	OR	95% CI
Household level	Non-index	15/1,055 (1.4)	1		1	
	Index	19/509 (3.7)	2.0	0.7–5.9	1.39	0.5–3.6
Distance from index case household (m) (17 missing)	0	19/512 (3.7)	1		1	
	0.1–149	2/258 (0.8)	0.29	0.1–1.7	0.28	0.03–2.7
	150+	13/777 (1.7)	0.60	0.2–1.8	-	-
Time from index case diagnosis (days)	0–7	12/639 (1.9)	1		1	
	8–14	0/156 (0.0)	-	-	-	-
	15–28	3/302 (1.0)	0.52	0.1–2.2	0.21	0.03–1.3
	28+	19/467 (4.1)	1.35	0.4–4.1	1.85	0.6–5.5
**Index Case**						
Sex	Female	8/421 (1.9)	1		1	
	Male	26/1,143 (2.3)	0.80	0.3–2.4	0.76	0.3–2.1
Age category (years)	<5	2/150 (1.3)	1		1	
(27 missing)	5–14	2/289 (0.7)	0.46	0.05–4.2	0.30	0.03–2.5
	15–24	25/586 (4.3)	2.43	0.4–13.2	1.4	0.3–7.5
	25–34	0/232 (0.0)	-		-	-
	35–44	1/148 (0.7)	0.53	0.04–7.1	0.52	0.04–7.1
	45+	3/132 (2.3)	1.77	0.2–14.1	7.16	0.5–96.6
Season	Low	7/550 (1.3)	1		1	
	High	27/1,014 (2.7)	1.46	0.5–4.3	4.07	1.4–11.7*
Local/imported	Local	21/1,102 (1.9)	1		1	
	Imported	13/462 (2.8)	0.43	0.1–1.8	1.51	0.4–5.4
Used a bednet (29 missing)	No	32/1,260 (2.5)	1		1	
	Yes	2/275 (0.7)	0.36	0.1–1.9	0.04	0.01–0.9*
House sprayed	No	27/1,036 (2.6)	1		1	
(57 missing)	Yes	7/471 (1.5)	0.78	0.3–2.3	0.24	0.05–1.2
**Individual**						
Sex	Female	18/824 (2.1)	1		1	
(3 missing)	Male	16/722 (2.2)	0.40	0.1–1.1	0.39	0.1–1.1
Age category (years)	<5	5/238 (2.1)	1		1	
(7 missing)	5–14	8/430 (1.9)	0.76	0.2–2.5	0.74	0.2–2.4
	15–24	15/447 (3.4)	0.67	0.2–2.5	0.62	0.2–2.3
	25–34	1/155 (0.7)	0.25	0.02–2.3	0.27	0.03–2.4
	35–44	1/98 (1.0)	0.40	0.04–3.9	0.51	0.06–4.6
	45+	4/189 (2.1)	0.92	0.2–3.8	0.57	0.1–2.5
Travelled to Angola	No	34/1,516 (2.2)	1		1	
	Yes	0/48 (0.0)	-	-	-	-
Used a bednet	No	30/1,190 (2.5)	1		1	
(12 missing)	Yes	4/362 (1.1)	0.50	0.2–1.5	0.57	0.8–1.8
House sprayed	No	26/1,060 (2.5)	1		1	
(72 missing)	Yes	8/432 (1.9)	1.14	0.4–3.3	2.82	0.9–9.3

OR: odds ratio; CI: confidence interval; m: meters

* p-value <0.05

### RDT sensitivity

Of the 47 secondary infections detected by LAMP in both case and control neighbourhoods, only 7 (15%) were detected by RDT. When stratified by case or control neighbourhood, estimates of diagnostic performance were fairly similar, with low sensitivity and positive predictive value and high specificity and negative predictive value (Table 4). A high proportion of RDT positive individuals screened during RACD were negative by LAMP in case (n = 5; 45%), neighbors of case (n = 6; 86%) and control (n = 5; 100%) households.

**Table 4:** Performance of RDTs using LAMP as gold standard in case and control neighbourhoods.

Neighbourhood	Sensitivity	Specificity	PPV	NPV
Case	17% (7/42)	99% (1,803/1,814)	39% (7/18)	98% (1,803/1,838)
Control	0% (0/5)	99% (1,285/1,290)	0% (0/5)	99% (1,285/1,290)
Overall	15% (7/47)	99% (3,088/3,104)	30% (7/23)	99% (3,088/3,128)

Despite the very low sensitivity of RDTs, it was estimated that RACD using RDTs to screen 100% of the survey population would prevent 29–50% of the infections to mosquitoes. This relatively high impact from treating a small proportion of the infections is due to the higher infectiousness of patent to sub-patent infections. Conversely and importantly, undetected infections by RDT may be responsible for 50–71% of transmission from humans to mosquitoes.

## Discussion

Detection and treatment of the infectious reservoir is critical to the success of malaria elimination campaigns [18–21]. This case-control study found that malaria infections were clustered around passively detected cases and that RDTs identified only a small fraction (15%) of secondary infections. While sub-patent infections are likely to be lower density and less infectious to mosquitos, the large size of this reservoir suggests a role in ongoing transmission. Based on published estimates of patent and sub-patent infectiousness, we estimate that RACD using RDTs in this setting would prevent less than half (29–50%) of all infections to mosquitoes (based on LAMP positive infections). In order to successfully eliminate the infectious reservoir, it is clear that alternative, more aggressive, approaches to treating the parasite reservoir are required.

Our findings provide clear evidence that infections cluster around index cases in this area of Namibia, with a higher prevalence of secondary infections found around passively detected cases than in randomly selected control neighborhoods, i.e. places where no case had been reported. This translated to a six-fold increase in the odds of infection within case households compared to controls. This finding is in line with evidence from other parts of southern Africa, including a study in Zambia which found a higher prevalence of infection by PCR in case households compared to randomly-selected control households [8]. In Engela, neighbors of index cases also had a five-fold increase in the odds of secondary infection compared to control households, suggesting that screening the four nearest neighboring households did not reach the edge of the high risk cluster. A recent pooled analysis of RACD studies, found that sharing the household with an index case was associated with a five-fold increase in the prevalence of infection compared to neighboring households [6]. While there was a higher prevalence of infection in index case households compared to neighboring households (3.4% vs 1.4%) in our study, the difference did not reach statistical significance with the sample size available. Together, these findings confirm that while risk is likely to be highest within index case households, the excess risk in neighboring households compared to controls suggests a wider screening radius around index cases might be appropriate in this context.

Our study did show limited evidence of a protective effect of vector control in reducing the odds of secondary infections occurring. Individuals who reported sleeping under a net had lower, but non-significant, odds of secondary infection. In addition, the odds of secondary infection was lower within the neighborhoods of index cases that reported sleeping under a net and/or in a sprayed structure, compared to case neighborhoods that had not reported any vector control intervention. The lack of statistical significance could be due to small sample size and the overall low coverage of vector control interventions.

There are several limitations to the case-control study that stem, in part, from operational challenges of conducting RACD in this setting. First, coverage of eligible cases was estimated to be low (50.7%) as described in a previous publication [12]. It is likely that coverage was lowest during the high transmission season, due to greater case burden, which may lead to under-representation of seasonal risk factors in the data. Second, a high proportion of individuals did not have a blood sample (19.6%) and nearly a third of those missing were attributed to lack of consent. Reasons why individuals refused to participate were not documented, but other ongoing studies in northern Namibia have found refusal rates for RACD to be lower and associated mainly with feeling well, repeat testing and lack of time. Third, a number of individuals were excluded from the study due to missing data. While absenteeism was relatively low in case (9.8%) and control (12.1%) neighborhoods, blood samples were not available for a higher proportion of individuals (12.0% and 28.5%). Data deficiencies could introduce bias into prevalence estimates and risk factor estimates if data are missing not at random (i.e. the probability of being missing is related to the risk of secondary infection). In this case, we did find that absent individuals were more likely to be male than those present, which could bias estimates of gender-related risk factors and potentially affect prevalence estimates in case and control neighborhoods. Finally, measurement error in GPS using tablets to calculate distance from the index case, may have obscured the ability to detect clustering at such small scales. Any such error is likely to introduce noise, and underestimate true associations, but is unlikely to bias the results.

This study has provided evidence that secondary infections cluster around index cases in Namibia and the low diagnostic sensitivity of standard RDTs critically limit the use of RACD to identify and treat infections. We estimate that RDTs had a sensitivity of only 15% compared to LAMP and that RACD treating only RDT positives would still allow 50–71% of infections to mosquitoes to occur in this setting. This is likely to be an underestimate, as it assumes 100% coverage of the target population, that LAMP misses no infections and that there are no treatment failures. Furthermore, this statistic only considers secondary infections within the target population, here defined as the four nearest neighbors (up to 30 individuals). Our results show that this screening radius is likely to be too small and will not capture all infections within the cluster. As all secondary infections identified by RDT and the majority (96%) identified by LAMP were afebrile, targeting reactive screening to people who report fever will be an ineffective strategy in this setting. The projected failure of RDTs to identify and treat a large portion of the malaria reservoir is consistent with studies that have found that RDTs detect only a small fraction of infections [22, 23], and those that demonstrated a lack of impact of RDT-based screening and treatment in Zanzibar, Burkina Faso and Kenya [11, 24–26].

As highlighted above, this research adds to a growing body of literature that reaches some common conclusions around the difficulties related to implementing RACD in the field. Diagnostic limitations aside, the considerable operational costs of RACD together with limited capacity of field teams to respond promptly to index cases due and achieve high coverage have been identified as key challenges in this and other studies [27, 28]. As stated in several publications, these limitations make RACD unlikely to be successful where the objective is to identify and treat every last case to eliminate transmission [27, 28]. However, there are other reasons that programmes may wish to use RACD, including to prevent outbreaks and to map out microscale patterns of transmission to provide detailed information where transmission is most likely to be occurring and improve further targeting of interventions [29].

Our results provide evidence that more sensitive field friendly diagnostics, or the use of presumptive treatment, may be required to interrupt transmission. Targeted vector control might also be a useful accompanying strategy to address limitations with interventions solely focused on the parasite. Reactively screening people with more sensitive diagnostics who travel or work together may be a more successful strategy where transmission occurs away from the home, as in Cambodia [5] or northern Senegal [30]. These approaches are currently under investigation in Indonesia. RACD is well-recognized to be a challenging activity, which ultimately needs to balance between operational and epidemiological considerations. Further operational research to refine this approach, not just in terms of radius but how to make it more epidemiologically targeted and efficient, is critical for its continued use in the field.

## Conclusions

Identifying and targeting the infectious reservoir is deemed critical to the success of malaria elimination campaigns. Malaria infections clustered in the household of passively detected cases and amongst their neighbors and RDTs identified only a small fraction (15%) of secondary infections. In order to successfully identify and eliminate the infectious reservoir, it is clear that alternative, more aggressive, approaches are required.

## References

[1] Sturrock HJW, Novotny JM, Kunene S, Dlamini S, Zulu Z, Cohen JM, et al Reactive Case Detection for Malaria Elimination: Real-Life Experience from an Ongoing Program in Swaziland. PloS one. 2013;8(5):e63830 doi: 10.1371/journal.pone.0063830 23700437 10.1371/journal.pone.0063830 PMC3658965

[2] Sturrock HJW, Hsiang MS, Cohen JM, Smith DL, Greenhouse B, Bousema T, et al Targeting Asymptomatic Malaria Infections: Active Surveillance in Control and Elimination. PLoS Med. 2013;10(6):e1001467 doi: 10.1371/journal.pmed.1001467 23853551 10.1371/journal.pmed.1001467 PMC3708701

[3] Stresman G, Kamanga A, Moono P, Hamapumbu H, Mharakurwa S, Kobayashi T, et al A method of active case detection to target reservoirs of asymptomatic malaria and gametocyte carriers in a rural area in Southern Province, Zambia. Malaria Journal. 2010;9(1):265 doi: 10.1186/1475-2875-9-265 20920328 10.1186/1475-2875-9-265 PMC2959066

[4] Bousema T, Griffin J, Sauerwein R, Smith D, Churcher T, Takken W, et al Hitting hotspots: spatial targeting of malaria for control and elimination. PLoS Med. 2012;9:e1001165 doi: 10.1371/journal.pmed.1001165 22303287 10.1371/journal.pmed.1001165 PMC3269430

[5] Hustedt J, Canavati SE, Rang C, Ashton RA, Khim N, Berne L, et al Reactive case-detection of malaria in Pailin Province, Western Cambodia: lessons from a year-long evaluation in a pre-elimination setting. Malaria journal. 2016;15(1):132 Epub 2016/03/05. doi: 10.1186/s12936-016-1191-z ; PubMed Central PMCID: PMC4774174. 26931488 10.1186/s12936-016-1191-z PMC4774174

[6] van Eijk AM, Ramanathapuram L, Sutton PL, Kanagaraj D, Sri Lakshmi Priya G, Ravishankaran S, et al What is the value of reactive case detection in malaria control? A case-study in India and a systematic review. Malaria journal. 2016;15(1):67 Epub 2016/02/08. doi: 10.1186/s12936-016-1120-1 ; PubMed Central PMCID: PMC4744450. 26852118 10.1186/s12936-016-1120-1 PMC4744450

[7] Okell L, Bousema T, Giffin J, Ouedraogo A, Ghani A, Drakeley C. Factors determining the occurrence of submicroscopic malaria infections and their relevance for control. Nat Commun. 2012;3:1237 doi: 10.1038/ncomms2241 23212366 10.1038/ncomms2241 PMC3535331

[8] Stresman GH, Kamanga A, Moono P, Hamapumbu H, Mharakurwa S, Kobayashi T, et al A method of active case detection to target reservoirs of asymptomatic malaria and gametocyte carriers in a rural area in Southern Province, Zambia. Malaria journal. 2010;9:265 Epub 2010/10/06. doi: 10.1186/1475-2875-9-265 ; PubMed Central PMCID: PMCPMC2959066. 20920328 10.1186/1475-2875-9-265 PMC2959066

[9] Tadesse FG, Pett H, Baidjoe A, Lanke K, Grignard L, Sutherland C, et al Submicroscopic carriage of Plasmodium falciparum and Plasmodium vivax in a low endemic area in Ethiopia where no parasitaemia was detected by microscopy or rapid diagnostic test. Malaria journal. 2015;14:303 Epub 2015/08/06. doi: 10.1186/s12936-015-0821-1 ; PubMed Central PMCID: PMC4524028. 26242243 10.1186/s12936-015-0821-1 PMC4524028

[10] Bousema T, Okell L, Felger I, Drakeley C. Asymptomatic malaria infections: detectability, transmissibility and public health relevance. Nature reviews Microbiology. 2014;12(12):833–40. Epub 2014/10/21. doi: 10.1038/nrmicro3364 . 25329408 10.1038/nrmicro3364

[11] Cook J, Xu W, Msellem M, Vonk M, Bergstrom B, Gosling R, et al Mass screening and treatment on the basis of results of a Plasmodium falciparum-specific rapid diagnostic test did not reduce malaria incidence in Zanzibar. The Journal of infectious diseases. 2015;211(9):1476–83. Epub 2014/11/28. doi: 10.1093/infdis/jiu655 . 25429102 10.1093/infdis/jiu655 PMC10881232

[12] Smith JL, Auala J, Haindongo E, Uusiku P, Gosling R, Kleinschmidt I, et al Malaria risk in young male travelers but local transmission persists: A case-control study in low transmission Namibia. Malaria journal. 2017;(accepted). 10.1186/s12936-017-1719-x PMC5303241 28187770

[13] Tambo M, Auala J, Sturrock HJ, Kleinschmidt I, Bock R, Smith JL, et al Evaluation of Loop-mediated isothermal Amplification as a Surveillance Tool For Malaria in Reactive Case Detection moving towards elimination. 2017. 10.1186/s12936-018-2399-x PMC6038281 29986717

[14] World Health Organization. Disease surveillance for malaria elimination: an operational manual. Geneva, Switzerland: 2012.

[15] R Core Team. R: A language and environment for statistical computing. Vienne, Australia: 2016.

[16] Okell LC, Bousema T, Griffin JT, Ouedraogo AL, Ghani AC, Drakeley CJ. Factors determining the occurrence of submicroscopic malaria infections and their relevance for control. Nature communications. 2012;3:1237 Epub 2012/12/06. doi: 10.1038/ncomms2241 ; PubMed Central PMCID: PMC3535331. 23212366 10.1038/ncomms2241 PMC3535331

[17] Mappin B, Cameron E, Dalrymple U, Weiss DJ, Bisanzio D, Bhatt S, et al Standardizing Plasmodium falciparum infection prevalence measured via microscopy versus rapid diagnostic test. Malaria journal. 2015;14:460 Epub 2015/11/19. doi: 10.1186/s12936-015-0984-9 ; PubMed Central PMCID: PMC4650290. 26577805 10.1186/s12936-015-0984-9 PMC4650290

[18] Wu L, van den Hoogen LL, Slater H, Walker PG, Ghani AC, Drakeley CJ, et al Comparison of diagnostics for the detection of asymptomatic Plasmodium falciparum infections to inform control and elimination strategies. Nature. 2015;528(7580):S86–93. Epub 2015/12/04. doi: 10.1038/nature16039 . 26633770 10.1038/nature16039

[19] The mal ERACGoD, Diagnostics. A Research Agenda for Malaria Eradication: Diagnoses and Diagnostics. PLoS medicine. 2011;8(1):e1000396 doi: 10.1371/journal.pmed.1000396 21311583 10.1371/journal.pmed.1000396 PMC3026696

[20] Tietje K, Hawkins K, Clerk C, Ebels K, McGray S, Crudder C, et al The essential role of infection-detection technologies for malaria elimination and eradication. Trends in parasitology. 2014;30(5):259–66. Epub 2014/04/15. doi: 10.1016/j.pt.2014.03.003 . 24726857 10.1016/j.pt.2014.03.003

[21] Gerardin J, Ouedraogo AL, McCarthy KA, Eckhoff PA, Wenger EA. Characterization of the infectious reservoir of malaria with an agent-based model calibrated to age-stratified parasite densities and infectiousness. Malaria journal. 2015;14:231 Epub 2015/06/04. doi: 10.1186/s12936-015-0751-y ; PubMed Central PMCID: PMC4702301. 26037226 10.1186/s12936-015-0751-y PMC4702301

[22] Gerardin J, Bever CA, Hamainza B, Miller JM, Eckhoff PA, Wenger EA. Optimal Population-Level Infection Detection Strategies for Malaria Control and Elimination in a Spatial Model of Malaria Transmission. PLoS Computational Biology. 2016;12(1):e1004707 doi: 10.1371/journal.pcbi.1004707 26764905 10.1371/journal.pcbi.1004707 PMC4713231

[23] Slater HC, Ross A, Ouedraogo AL, White LJ, Nguon C, Walker PG, et al Assessing the impact of next-generation rapid diagnostic tests on Plasmodium falciparum malaria elimination strategies. Nature. 2015;528(7580):S94–101. Epub 2015/12/04. doi: 10.1038/nature16040 . 26633771 10.1038/nature16040

[24] Halliday KE, Okello G, Turner EL, Njagi K, McHaro C, Kengo J, et al Impact of intermittent screening and treatment for malaria among school children in Kenya: a cluster randomised trial. PLoS medicine. 2014;11(1):e1001594 Epub 2014/02/05. doi: 10.1371/journal.pmed.1001594 ; PubMed Central PMCID: PMC3904819. 24492859 10.1371/journal.pmed.1001594 PMC3904819

[25] Tiono AB, Ouedraogo A, Ogutu B, Diarra A, Coulibaly S, Gansane A, et al A controlled, parallel, cluster-randomized trial of community-wide screening and treatment of asymptomatic carriers of Plasmodium falciparum in Burkina Faso. Malaria journal. 2013;12:79 Epub 2013/02/28. doi: 10.1186/1475-2875-12-79 ; PubMed Central PMCID: PMC3599538. 23442748 10.1186/1475-2875-12-79 PMC3599538

[26] von Seidlein L. The Failure of Screening and Treating as a Malaria Elimination Strategy. PLoS medicine. 2014;11(1):e1001595 doi: 10.1371/journal.pmed.1001595 24492211 10.1371/journal.pmed.1001595 PMC3904824

[27] Searle KM, Hamapumbu H, Lubinda J, Shields TM, Pinchoff J, Kobayashi T, et al Evaluation of the operational challenges in implementing reactive screen-and-treat and implications of reactive case detection strategies for malaria elimination in a region of low transmission in southern Zambia. Malaria journal. 2016;15(1):412 Epub 2016/08/17. doi: 10.1186/s12936-016-1460-x ; PubMed Central PMCID: PMC4986207. 27527347 10.1186/s12936-016-1460-x PMC4986207

[28] Donald W, Pasay C, Guintran JO, Iata H, Anderson K, Nausien J, et al The Utility of Malaria Rapid Diagnostic Tests as a Tool in Enhanced Surveillance for Malaria Elimination in Vanuatu. PloS one. 2016;11(11):e0167136 Epub 2016/12/03. doi: 10.1371/journal.pone.0167136 ; PubMed Central PMCID: PMCPMC5130254. 27902755 10.1371/journal.pone.0167136 PMC5130254

[29] Larsen DA, Ngwenya-Kangombe T, Cheelo S, Hamainza B, Miller J, Winters A, et al Location, location, location: environmental factors better predict malaria-positive individuals during reactive case detection than index case demographics in Southern Province, Zambia. Malaria journal. 2017;16(1):18 Epub 2017/01/08. doi: 10.1186/s12936-016-1649-z ; PubMed Central PMCID: PMCPMC5219724. 28061853 10.1186/s12936-016-1649-z PMC5219724

[30] Littrell M, Sow GD, Ngom A, Ba M, Mboup BM, Dieye Y, et al Case investigation and reactive case detection for malaria elimination in northern Senegal. Malaria journal. 2013;12:331 Epub 2013/09/21. doi: 10.1186/1475-2875-12-331 ; PubMed Central PMCID: PMC3848815. 24044506 10.1186/1475-2875-12-331 PMC3848815

